# Investigating the Impact of Origins on the Quality Characteristics of Celery Seeds Based on Metabolite Analysis through HS-GC-IMS, HS-SPME-GC-MS and UPLC-ESI-MS/MS

**DOI:** 10.3390/foods13101428

**Published:** 2024-05-07

**Authors:** Jun Yan, Lizhong He, Zhiwu Huang, Hong Wang, Li Yu, Weimin Zhu

**Affiliations:** Horticulture Research Institute, Shanghai Academy of Agricultural Sciences, Key Laboratory of Protected Horticulture Technology, Shanghai 201403, China; ylgw_07@126.com (J.Y.); yuhe_5@126.com (L.Y.)

**Keywords:** celery seed, volatile metabolites, non-volatile metabolites, metabolomics

## Abstract

Celery seeds contain various bioactive compounds and are commonly used as a spice and nutritional supplement in people’s daily lives. The quality of celery seeds sold on the market varies, and their regions of production are unclear. This study evaluated the metabolites of Chinese celery seeds from three production regions using HS-SPME-GC-MS, HS-GC-IMS, and UPLC-ESI-MS/MS. The results indicate that GC-IMS analysis obtained a metabolic profile different from that detected using GC-MS. Terpenoids, polyphenols, coumarins, and phthalides are the main bioactive compounds in celery seeds. The production region significantly affects the metabolic characteristics of celery seeds. Based on GC-MS data, GC-IMS data, and LC-MS data, the variation analysis screened 6, 12, and 8 metabolites as potential characteristic metabolites in celery seeds related to the production region, respectively. According to the aromatic characteristics of the characteristic metabolites, seeds from the HCQ region and HZC region have a strong herbal, woody, celery, and turpentine aroma. The concentration of secondary metabolites was highest in the seeds from the HCQ region followed by the HZC region, and it was the lowest in the JJC region. Altogether, this study investigates how geographical origins influence the metabolomic profile of celery seeds. The results can be used to guide the planting and harvesting of celery seeds in suitable regions.

## 1. Introduction

Celery (*Apium graveolens* L.) is a popular vegetable globally. Its aromatic fruits (called celery seeds) are elliptical, 1.5–2 mm in size, and brown in color with black ridges. Celery seeds are used as a spice to add a distinctive texture and pungent taste to soups, salads, pickles, bakery products, and fish and meat dishes. Powders made from these seeds can also be mixed with table salt or pepper to be used as a condiment [1,2,3]. Numerous medical studies have shown that celery seeds have good medicinal value. For example, the seed extract can be used to treat hypertension, alleviate gout [4], prevent cardiovascular disease [5], prevent Alzheimer’s disease [6], and inhibit cancer cell growth [7]. Moreover, celery seed powder is an effective nutritional supplement. In China, people use ground celery seed powder to prevent diseases such as hypertension, arthritis, and gout. At present, research on the bioactive compounds in celery seeds mainly focuses on their medicinal mechanisms, with a few studies identifying and analyzing the aromatic compounds in celery seeds. Mostaphiet et al. [8], Xu et al. [9], and Yan et al. [10] used GC-MS technology to identify 12, 56, and 195 volatile compounds in celery seeds, respectively. Celery seeds contain not only volatile compounds but also many non-volatile secondary metabolites. LC-MS technology is widely used for analyzing plant metabolites [11]. Thus far, no comprehensive analysis has been conducted on the volatile and non-volatile metabolites in celery seeds.

The type and content of plant metabolites depend on various factors, such as the climate, soil, extraction methods, and extraction sites [12,13]. Using volatile metabolomics, Feng et al. [14] revealed remarkable metabolic differences in *Zanthoxylum bungeanum* from eight geographical regions and identified four and eight potential markers for distinguishing red and green *Z. bungeanum*, respectively. According to GC-IMS, Molixiang grapes from three geographical origins of China showed significant differences in their aromatic compounds [15]. Metabolomics revealed eight metabolites in Echinacea purpurea which were related to geographical location and environmental variables [12]. Our previous study found that the flavonoid content was significantly different in celery stalks originating from different regions of China [16]. Research on the essential oils of celery seeds found that their composition is related to the production region [3]. Understanding the differences in the bioactive components of celery seeds from different origins can guide farmers to plant and harvest celery seeds in specific geographical areas, thereby improving product quality. However, thus far, the impact of the production region on the variability in celery seed metabolites has not been reported.

With the increasing demand for healthier lifestyles, the consumption of celery seeds is constantly increasing. Currently, the quality of celery seeds sold on the market varies, and their regions of production are unclear. Metabolomics can reveal intra- and interspecies differences in plants grown in the same or different production regions [17]. Therefore, this study collected celery seeds from three major production regions of China and investigated their volatile compounds and non-volatile secondary metabolites using GC-IMS, GC-MS, and LC-MS. Based on multivariate statistical analysis, differences in the non-volatile and volatile metabolites in celery seeds from different production regions were analyzed to examine the differences in quality. The results provide insights into suitable planting areas for celery seed production and provide a basis for the geographical identification of celery seeds.

## 2. Materials and Methods

### 2.1. Plant Materials

**Figure 1:**
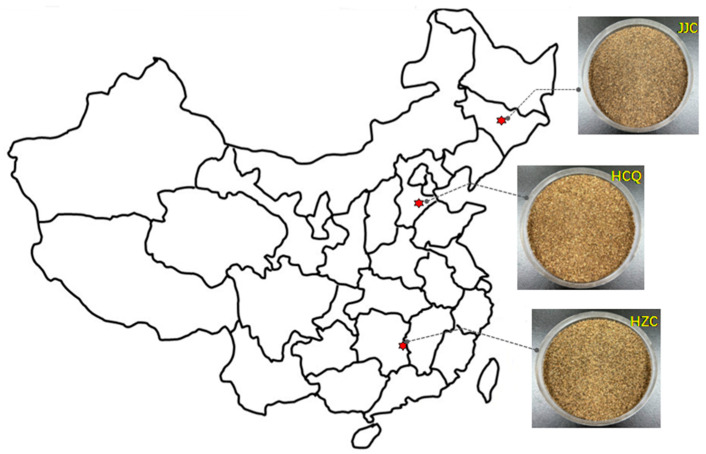
Distribution map of three celery seeds. HCQ = Cangzhou, Qingxian County, Hebei Province; HZC = Zhuzhou, Chaling County, Hunan Province, JJC: Chuanying District, Jilin, Jilin Province.

Celery seed samples of the “yellow heart” Chinese celery variety were collected from three regions in China: Cangzhou in Qingxian County of Hebei Province (HCQ); Zhuzhou in Chaling County of Hunan Province (HZC); and Chuanying District in the city of Jilin in Jilin Province (JJC) (Figure 1). Random sampling was conducted using a five-point sampling method within the cultivation area in June 2022. Detailed information on the cultivation areas is presented in Table S1. Cleaned and dried (humidity: 8–9%) seed samples were ground into powder and then passed through 40 mesh sieves (850 µm ± 29 µm) and stored at −80 °C until needed.

### 2.2. Headspace-GC-IMS Analysis

Volatile metabolites were detected using a GC-IMS system. The celery seed powders (1 g) were transferred into a 20 mL headspace bottle and incubated at 40 °C (stirring speed: 500 rpm) for 20 min. Then, 500 μL of the headspace gas was injected into the injection port at 85 °C. Volatile metabolites were separated using an FS-SE-54 chromatographic column (15 m × 0.53 mm) at 60 °C. The initial flow rate of the carrier gas (high-purity nitrogen (>99.9%)) was 2 mL/min for 2 min, which then increased to 10 mL/min at 10 min and 100 mL/min at 20 min and then was maintained for 40 min. Subsequently, the pre-separated volatiles were ionized using a 3H ionization source (5 kV). Thereafter, the ionized volatiles were transferred into a drift tube full of nitrogen (>99.9% purity) at 45 °C (flow rate of 150 mL/min). Finally, based on the different migration rates, the volatile metabolites were separated and identified by searching the National Institute of Standards and Technology (NIST) library and the IMS database from G.A.S. (Dortmund, Germany) using the retention index (RI) and drift time (Dt). The volatile metabolite content was presented as the signal intensity. Two-dimensional top views and the volatile metabolites’ fingerprints were generated by the two plugins (Reporter and Gallery Plot) in the GC-IMS system.

### 2.3. Headspace Solid-Phase Microextraction GC–MS (HS-SPME-GC-MS) Analysis

For HS-SPME-GC-MS analysis, 500 mg of powder from each variety of celery seed was poured into a 20 mL headspace bottle containing NaCl saturated solution and 10 μL (50 μg/mL) 3-Hexanone (internal standard). The bottles were kept at 60 °C for 5 min and then absorbed by a divinylbenzene/carbon wide-range/polydimethylsiloxane fiber (DVB/CWR/PDMS, 1.1 mm × 120 μm, Agilent J &W Scientific, Folsom, CA, USA) for 15 min at 60 °C. Before GC-MS analysis, the solid-phase microextraction (SPME) fiber was desorbed at 250 °C for 5 min. The volatile metabolites were detected using an Agilent Model 8890 GC and a 7000D mass spectrometer (Agilent). The carrier gas was helium with a linear velocity of 1.2 mL/min. The capillary column (DB-5MS, 30 m × 0.25 mm × 0.25 μm) temperature was initially 60 °C, which was maintained for 3.5 min, and it was heated to 100 °C at a rate of 10 °C/min, to 180 °C at a rate of 7 °C/min, and to 280 °C at a rate of 25 °C/min, at which it was held for 5 min. The quadrupole mass detector and ion source temperatures were 150 and 230 °C, respectively. The ion source was operated in electron impact (EI) mode at 70 eV. The full scan mode was used at 40–400 *m*/*z*. The unknown volatile metabolites were identified by searching the retention index (RI) in the NIST 2018 software data. The relative content of volatile metabolites was quantified based on their peak areas.

### 2.4. UPLC-ESI-MS/MS Analysis

The celery seed powder (50 mg) was transferred into a methanol solution (1.2 mL, 70%) by eddying for 30 s every 30 min 6 times. Then, the mixture was centrifuged at 12,000 rpm for 3 min at 4 °C, and the supernatant was passed through a 0.22 μm filter membrane before LC-MS detection.

The non-volatile metabolites were detected using a UPLC-ESI-MS/MS system (UPLC, SHIMADZU Nexera X2; MS, Applied Biosystems 6500 Q TRAP, shimadzu, Kyoto, Japan) equipped with an Agilent SB-C18 chromatographic column (1.8 µm, 2.1 mm × 100 mm). The LC conditions were as follows: mobile phase A, containing pure water with 0.1% formic acid; mobile phase B, containing acetonitrile with 0.1% formic acid; a column temperature of 40 °C; and an injection volume of 4 μL. The gradient elution program was as follows: 0.00 min, 5% B; 9.00 min, 5–95% B; 1 min, 95% B; 11.10 min, 95–5% B; and 14 min, 5% B, with a flow rate of 0.35 mL/min. The MS conditions were as follows: an ion spray voltage (IS) of 5500 V (positive ion mode) and 4500 V (negative ion mode); an electrospray ionization (ESI) temperature of 550 °C; and ion source gas I (GSI), gas II (GSII), and curtain gas (CUR) pressures of 50, 60, and 25 psi, respectively.

Based on the MVDB V 2.0 database from Maiwei (Wuhan) Biotechnology Co., Ltd., Wuhan, China, qualitative analysis of the metabolites was carried out using Analyst 1.6.3. software. Isotope signals, duplicate signals containing K+ ions, Na+ ions, and NH4+ ions, and duplicate signals of fragment ions which themselves were larger molecular weight substances were removed. Subsequently, the multiple reaction monitoring mode (MRM) was used for quantification. Peak area integration was performed on all mass spectra peaks of the metabolites, and the mass spectra peaks for the same metabolite in different samples were integrated and corrected. The area of each peak represents the relative content of the corresponding metabolite [18].

### 2.5. Data Analysis

Significant differences among the metabolites were analyzed by one-way analysis of variance (ANOVA) using Duncan’s multiple range test (*p* < 0.05) with SPSS 25.0 software. Principle component analysis (PCA) and orthogonal partial least squares discriminant analysis (OPLS-DA) were performed using SIMCA software 14. Heat maps were generated using MetabAnalyst 5.0.

## 3. Results and Discussion

### 3.1. Qualitative Analysis of Volatile Metabolites of Celery Seeds

To comprehensively and accurately analyze the aromatic composition of celery seeds, HS-GC-IMS and HS-SPME-GC-MS were used to identify the volatile metabolites present in celery seeds from different production regions. HS-SPME-GC-MS analysis identified 147 volatile metabolites in the celery seed samples from three geographical origins (Table S2). The total ion chromatogam (TIC) of the QC sample (quality control samples prepared by mixing samples) is shown in Figure S1. These volatile metabolites included 64 terpenoids, 21 alcohols, 20 esters, 12 aldehydes, 6 acids, 8 ketones, 5 phthalides, and 11 other compounds (Figure S2A). According to the peak area of the volatile metabolites, limonene, pinene, β-guaiene, isolepidozene, β-myrcene, β-phellandrene, (−)-zingiberene, 3-ethyl-2-methyl-1, 3-hexadiene, and aristolochene were the major volatile metabolites present in the celery seeds. Mostaphiet et al. [8], Xu et al. [9], and Yan et al. [10] showed that terpenoids are the most diverse type of volatile metabolites in celery seeds, which is consistent with our results.

**Figure 2:**
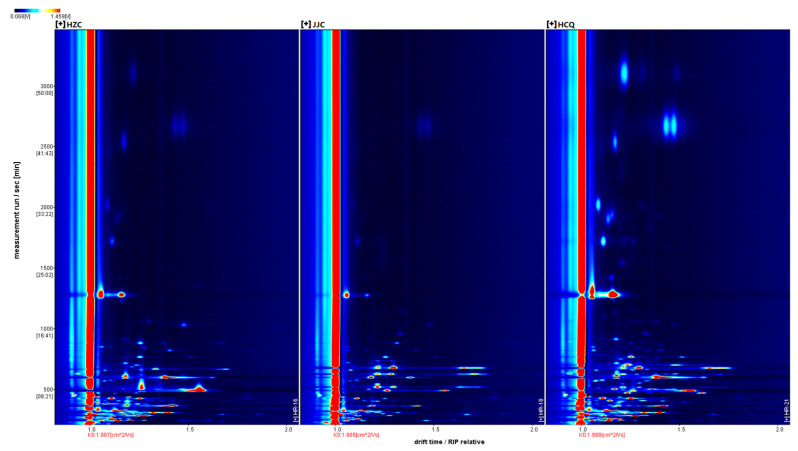
Two-dimensional chromatogram results of volatile metabolites in celery seeds from three production regions. The *X* axis represents the ion migration time, and the *Y* axis represents the gas chromatography retention time. The red vertical line at abscissa 1.0 represents the reaction ion peak (after normalization). Each point on either side of the ion peak represents a volatile metabolite. Red represents high signal intensity, and blue represents low signal intensity.

GC-IMS is an emerging technology that combines ion mobility spectroscopy with gas chromatography. GC-IMS does not require the concentrations of volatile metabolites in the sample through SPME; rather, it directly absorbs and detects the gas with a certain volume from the sample [19], which in addition to potentially detecting the true aromatic components of the sample more accurately is also faster, more sensitive, and more convenient than GC-MS [20]. Figure 2 shows a two-dimensional top view of the volatile metabolites in the celery seed samples obtained via GC-IMS. This two-dimensional top view shows that the types of volatile metabolites in the three celery seed samples were quite similar but with varying signal intensities. Based on the NIST2014 and IMS databases, 92 volatile metabolites were identified (Table S3). The monomeric and dimeric forms of 14 volatile metabolites were detected, including α-terpinene, methyl 2-methylbutanoate, and methyl 2-methylbutanoate. The monomeric, dimeric, and trimeric forms of beta-pinene, myrcene, and limonene were detected. In total, 72 volatile metabolites were identified in the celery seed samples, including 19 aldehydes, 11 terpenoids, 11 esters, 6 ketones, 8 alcohols, 2 acids, 7 other classes, and 8 unknown metabolites (Figure S2B). Compared with the GC-MS analysis, GC-IMS detected fewer volatile metabolites, and most had low RI values. It has been reported that GC-IMS is more sensitive to volatile metabolites with low melting points and is able to detect many small molecules and volatile components with low contents [21]. GC-IMS analysis identified volatile metabolites (butyl acetate, 2-propanol, (E)-2-Hexenal, etc.) in the form of monomers and dimers, as well as volatile metabolites that were not detected using HS-SPME-GC-MS (anisole, thiophene, Dimethyl sulfide, etc.) (Table S3). These varying results may be due to the different detection methods and sample pretreatment methods of these two instruments. This study is the first to use GC-IMS to detect volatile substances in celery seeds, obtaining a metabolic profile different from that found using GC-MS and providing data information for the rapid detection of volatile substances in celery seeds and related products in the future.

### 3.2. Qualitative Analysis of Non-Volatile Metabolites of Celery Seeds

A total of 383 non-volatile metabolites were detected in celery seeds from three different production regions using LC-MS analysis (Table S4), including 164 polyphenols, 45 lipids, 38 amino acids, 31 carbohydrates, 23 nucleosides, 22 organic acids, 8 vitamins, 6 terpenoids, 5 alkaloids, 8 phthalides, and 33 other compounds (Figure S2C). The total ion chromatogams (TICs) and MRM metabolite detection multi-peak chart of the QC sample is shown in Figures S3 and S4, respectively. In terms of quantity, the celery seed samples contained a large number of polyphenols (42.82% of 383), including 68 flavonoids, 67 phenolic acids, and 29 coumarins. These flavonoids included 10 apigenin and their derivatives, 8 luteolins and their derivatives, 4 quercetin derivatives, 4 chrysoeriol derivatives, 8 kaempferol derivatives, 5 naringenins and their derivatives, 4 diosmetins and their derivatives, 3 eriodictyols and their derivatives, 3 hispidulins and their derivatives, 2 galangins and their derivatives, and 2 dihydrokaempferol derivatives, as well as phenolic acids, namely 46 hydroxycinnamic acids and 21 hydroxybenzoic acids. Flavonoids, phenolic acids, and coumarins have been proven to have multiple medicinal functions. Compared with our previous detection of non-volatile metabolites in celery leaves [22], this study found that celery seeds have a greater variety of flavonoids and coumarins. Eight phthalides were detected, five of which were detected via GC-MS analysis. Phthalides contribute to the unique aroma of celery. Both the GC-MS and LC-MS methods are able to detect phthalides [23]. In addition, some terpenoids and alkaloids, as well as a variety of other secondary metabolites, were detected. A total of 45 lipids were detected in the celery seed samples, including 35 fatty acids, 6 glycerolesters, 1 sphingolipid, and 3 lysophosphatidylcholines. Basic amino acids, sugars, organic acids, etc. were also detected in celery seeds. We used LC-MS technology to analyze the secondary metabolites in celery leaves and detected a total of 127 polyphenols [22]. In this study, a total of 164 polyphenols were detected in the celery seeds, indicating that polyphenols are more abundant in celery seeds. We detected many metabolites that have not been previously reported in celery, such as eriodictyol and its derivatives, hispidulin and its derivatives, betaine, and trigonelline. This may be due to the differences in their concentrations in different tissues. Further experiments would be required to confirm the reliability of these metabolites’ detection. In a word, the above results lay the foundation for a comprehensive analysis of the metabolites of celery seeds and targeted development of related products.

### 3.3. Discriminating Celery Seeds from Different Geographical Origins via PCA

PCA, an unsupervised multivariate statistical analysis method, reveals the internal relationships between variables by transforming multiple variables into a few principal components, providing intuitive explanations for complex datasets and revealing the groupings and trends underlying the observed data in a dataset. It can also be used to select outlier samples [24]. In recent years, PCA has been proven to be able to effectively distinguish differences in crop origins and varieties [15].

**Figure 3:**
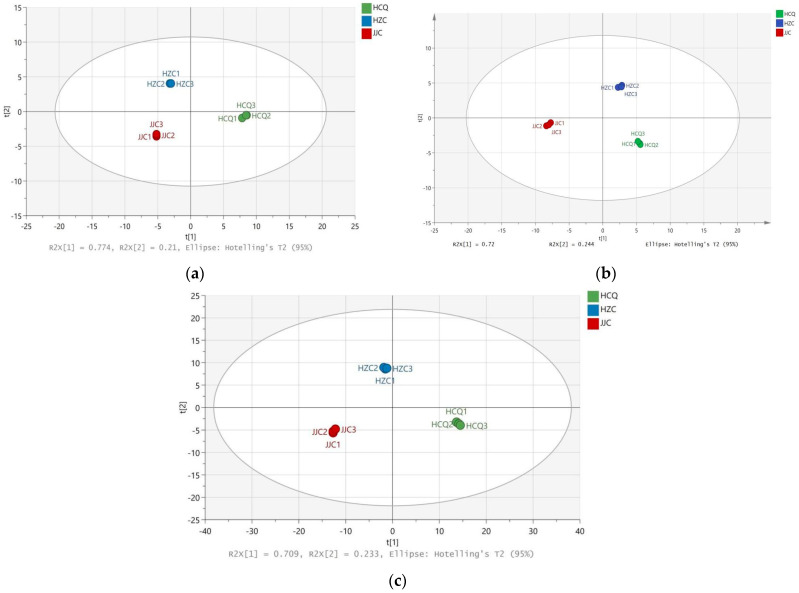
PCA score plot for celery seeds from three production regions based on GC-IMS data (**a**), GC-MS data (**b**), and LC-MS data (**c**).

To assess the discrimination among the celery seeds from different geographical origins, PCA was performed based on the GC-IMS data, GC-MS data, and LC-MS data. The PCA score plots are shown in Figure 3. The R2X results of the three PCA models were 0.983, 0.964, and 0.942, and those of Q2 were 0.974, 0.94, and 0.909, respectively. These results indicate that the three models had good fits. As shown in the three PCA score plots in Figure 3, the three groups of celery seed samples formed three independent areas. These results indicate that the production region significantly affects the metabolic characteristics and nutritional composition of celery seeds. Lau et al. [25] successfully distinguished celery specimens from three different regions using 1H NMR spectroscopy. Geographical sources have also been found to significantly affect the metabolic characteristics of plants such as black beans [11], Molixiang table grapes [15], and quinoa [26].

### 3.4. Variations in Volatile Metabolites of Celery Seeds from Different Geographical Origins

**Figure 4:**
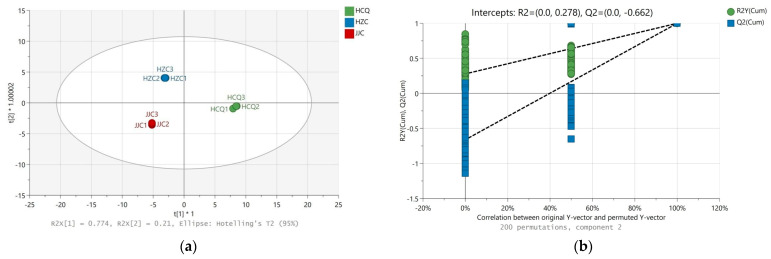
OPLS-DA score plot (**a**) and 200 permutation test (**b**) of volatile metabolites in celery seeds from three production regions based on GC-IMS data.

**Figure 5:**
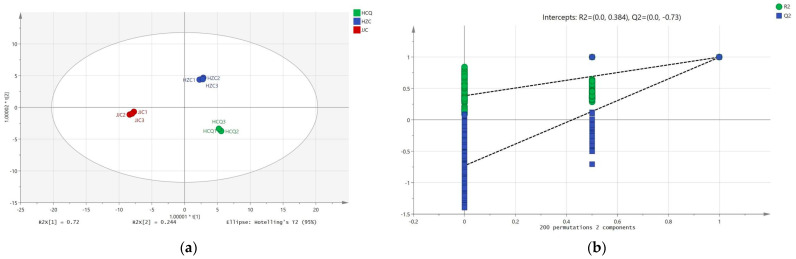
OPLS-DA score plot (**a**) and 200 permutation test (**b**) of volatile metabolites in celery seeds from three production regions based on GC-MS data.

OPLS-DA is a supervised pattern multivariate statistical analysis method that can be combined with orthogonal signal correction (OSC) to maximize inter-group differentiation. The projected variable of importance (VIP) value of the OPLS-DA model can be used to quantify the contribution of each variable to sample classification. To further analyze the differences in volatile metabolites in celery seeds from different geographical origins, OPLS-DA and one-way ANOVA were preformed based on GC-IMS and GC-MS data. In the OPLS-DA score plots using GC-IMS data (Figure 4a), the model parameters R2X, R2Y, and Q2 were 0.983, 0.999, and 0.998, respectively. The model parameters R2X, R2Y, and Q2 in the OPLS-DA score plots using the GC-MS data (Figure 5a) were 0.964, 0.998, and 0.996, respectively. Permutation tests were then conducted 200 times to determine whether the two OPLS-DA models were overfitted. As shown in Figure 4b and Figure 5b, the original R2Y and Q2Y values (the two rightmost points) were higher than the corresponding values after displacement on the left (the scattered points on the left), the R2Y intercept of the model was less than 0.5, and the intercept of Q2Y was negative. These results indicate that the two OPLS-DA models are reliable. Similar to the PCA, celery seeds from different regions can be clearly distinguished on the OPLS-DA score plots.

**Figure 6:**
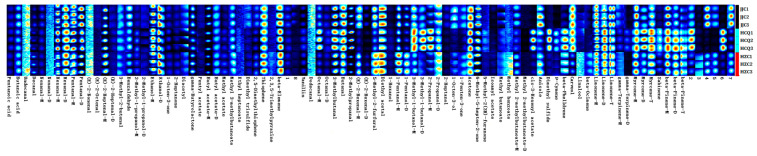
The volatile metabolite fingerprints of celery seed from three production regions generated by GC-IMS data.

**Figure 7:**
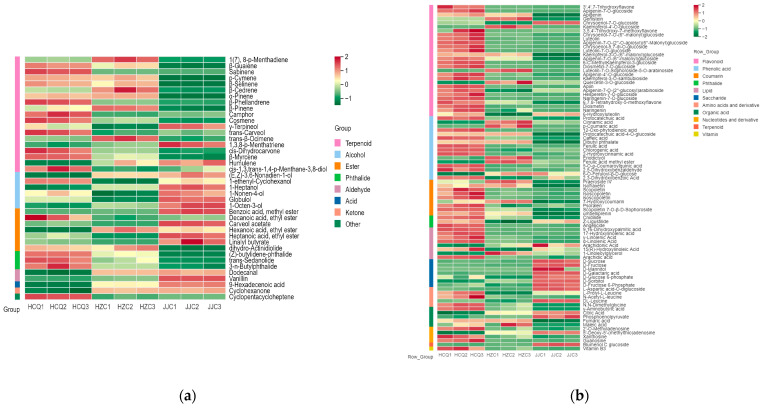
Heatmap of differential metabolites in celery seeds from three production regions detected via GC-MS (**a**) and LC-MS (**b**).

When combining the VIP values of the volatile metabolites from the OPLS-DA models and the one-way ANOVA, 34 and 40 volatile metabolites were identified as differential metabolites using GC-IMS and GC-MS (VIP > 1 and *p* < 0.05) (Tables S5 and S6), respectively. Xu et al. [27] found that the aromatic compounds present in five commercial celery seed essential oils were significantly different. In terms of quantity, terpenoids were the main differential metabolites. Phthalides are the source of celery’s special aroma. In China, 3-n-butylphthalide has been approved for the treatment of stroke patients [24]. The fingerprint spectra of GC-IMS data were generated using the Gallery Plot plugin (Figure 6). The fingerprint spectra of differential metabolites detected via GC-IMS (Figure 6) and the heatmaps of differential metabolites detected via CG-MS (Figure 7a) show that celery seeds from HCQ contained more terpenoids and phthalides. It was reported previously that terpenoids are positively linked to temperature and drought conditions [28]. Compared with the JJC region, the temperature was higher and precipitation was lower in the HCQ region during the celery seeds’ harvesting period (https://www.tianqi.com/ accessed on 10 June 2022). In a similar study, Park et al. [29] found that high cultivation temperatures increased the flavor components of sesame seeds.

In the GC-IMS OPLS-DA model, the VIP values of sabinene (odor: herbaceous and pine), p-cymene (odor: solvent, gasoline, and citrus), β-pinene (odor: pine, resin, and turpentine), β-phellandrene (odor: mint and turpentine), and two unidentified metabolites were all greater than two (Table S5), indicating that these metabolites might be characteristic volatile metabolites in celery related to the production region. In the GC-MS OPLS-DA model, the VIP values of β-selinene (odor: herbs), β-guaien (odor: wood and spice), sabinene (odor: herbs and pine), p-cymene (odor: solvent, gasoline, and citrus), 1-heptanol (odor: herbs, chemical, and green), cis-dihydrocarvone (odor: warm and herbs), humulene (odor: wood), methyl benzoate (odor: prune, lettuce, herb, and sweet), 3-n-butylphthalide (odor: celery), β-phellandrene (odor: mint and turpentine), (Z)-butylidene-phthalide (odor: celery), and β-pinene (odor: pine, resin, and turpentine) were all greater than two (Table S6), indicating that these metabolites might be characteristic volatile metabolites in celery related to the production region. Sabinene, p-cymene, β-pinene, and β-phellandrene exhibited higher VIP values in both models, indicating that the results were reliable. The relative content of these metabolites obtained from the two models were significantly higher in seeds from the HCQ region and HZC region than in seeds from the JJC region. According to the aromatic characteristics of these volatile metabolites, seeds from the HCQ region and HZC region have a strong herbal, woody, celery, and turpentine-like aroma.

### 3.5. Variations in Non-Volatile Metabolites of Celery Seeds from Different Geographical Origins

**Figure 8:**
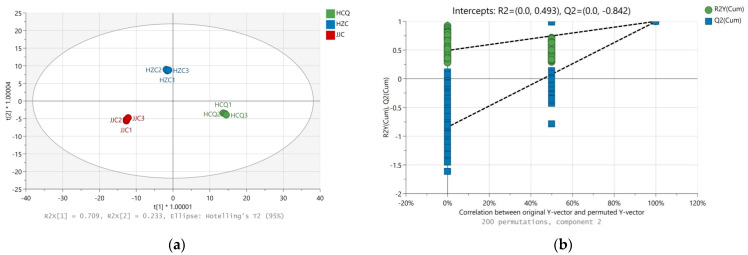
OPLS-DA score plot (**a**) and 200 permutation test (**b**) of volatile metabolites in celery seeds from three production regions based on LC-MS data.

Non-volatile metabolites in celery seeds include primary metabolites and secondary metabolites, which are closely related to celery seed growth and development. The bioactive compounds such as flavonoids, coumarins, and phenolic acids present in secondary metabolites are related to the medicinal value of celery seeds. Using VIP > 1 (OPLS-DA model) and *p* < 0.05 (ANOVA) as criteria, 87 differential non-volatile metabolites were identified in the celery seed samples from the three production origins (Table S7). The R2X, R2Y, and Q2 values for the OPLS-DA model were 0.942, 0.999, and 0.997, respectively. The results of 200 permutation tests proved that the OPLS-DA model was effective and reliable (Figure 8). In terms of quantity, the main differential metabolites were secondary metabolites, including flavonoids, phenolic acids, coumarins, and phthalides, which accounted for 64.36% of the differential metabolites. Flavonoids and phenolic acids have multiple medicinal effects. Among the different metabolites, apigenin and its derivatives, chrysoeriol and its derivatives, luteolin and its derivatives, cinnamic acid, p-coumaric acid, and chlorogenic acid have been demonstrated to have antioxidant, anti-inflammatory, anti-cancer activities, as well as preventive and therapeutic effects for cardiac, cerebral, and vascular diseases [30,31,32]. Coumarin has some biological activities, such as anti-tumor, anti-analgesic, and anti-depressive properties but can also easily cause photosensitive dermatitis [33]. In some traditional Chinese medicine treatments, furanocoumarins have been used for the photo-chemotherapy of psoriasis and atopic eczema [34]. In addition, α-linolenic acid has been proven to be effective at preventing non-alcoholic fatty liver disease [35]. As shown in Figure 7b, the concentration of secondary metabolites was highest in the celery seeds from the HCQ region followed by HZC, with the lowest concentration found in the JJC region. The climate of the production region, including the average temperature, precipitation, and light, is closely related to the accumulation of secondary metabolites in plants [36]. Usually, high temperatures and drought increase the accumulation of flavonoids and phenolic acids in plants such as corn [29], sorghum [37], and tea trees [38]. Compared with the JJC region, the temperature was higher and the precipitation was lower in the HCQ region during the celery seeds’ harvest period (https://www.tianqi.com/ accessed on 10 June 2022).

In the LC-MS OPLS-DA model, the VIP values of 30 differential metabolites were higher than two (Table S7), among which the VIP values of 3’,4’,7-trihydroxyflavone, apigenin, genistein, cinnamic acid, apigenin-7-O-glucoside, protocatechuic acid, p-coumaric acid, and chrysoeriol-7-O-glucoside were higher than three, indicating that these metabolites might be non-volatile characteristic metabolites in celery related to the production region. These characteristic metabolites are related to the medicinal function of celery seeds and can be used to evaluate the quality of celery seeds.

## 4. Conclusions

In this study, GC-IMS, GC-MS, and LC-MS techniques were used to determine the differences in the volatile and non-volatile metabolites of celery seeds from three geographical regions. The results indicated that terpenoids are the main volatile metabolites in celery seeds. GC-IMS was first used to analyze the volatile metabolites in celery seed, and it obtained a metabolic profile different from that detected using GC-MS. Compared with a previous study on celery leaves, celery seeds contain more secondary metabolites, mainly polyphenols (42.82% of all non-volatile metabolites). PCA and OPLS-DA of the metabolites obtained from three techniques revealed that the production region has a remarkable influence on the metabolic properties of celery seeds, evidenced by the celery seeds from three different regions having unique distributions in their score plots. Based on GC-IMS data, GC-MS data, and LC-MS data, 34, 40, and 87 differential metabolites were identified (VIP > 1 and *p* < 0.05), respectively. The GC-IMS OPLS-DA model and GC-MS OPLS-DA model screened 6 and 12 characteristic volatile metabolites related to the production region, respectively. Sabinene, p-cymene, β-pinene, and β-phellandrene were screened as characteristic volatile metabolites in both models. The relative contents of these characteristic volatile metabolites were significantly higher in seeds from the HCQ region and HZC region than in seeds from the JJC region. According to the aromatic characteristics of these metabolites, seeds from the HCQ region and HZC region have a strong herbal, woody, celery, and turpentine aroma. The LC-MS OPLS-DA model screened eight characteristic non-volatile metabolites, which were 3’,4’,7-trihydroxyflavone, apigenin, genistein, apigenin-7-O-glucoside, protocatechuic acid, chrysoeriol-7-O-glucoside, cinnamic acid, and p-coumaric acid. The concentration of secondary metabolites was the highest in the seeds from the HCQ region followed by the HZC region, and it was the lowest in the JJC region. In brief, the present study investigated how geographical origins influence the metabolomic profile of celery seeds. The results can be used to guide the planting and harvesting of celery seeds in suitable regions. The GC-IMS analysis provided data for the rapid detection of volatile substances in celery seeds and related products. In future research, we will study the biological activity of celery seeds from different production areas.

## Data Availability

The original contributions presented in the study are included in the article and supplementary materials, further inquiries can be directed to the corresponding author.

## Supplementary Materials

The following supporting information can be downloaded at https://www.mdpi.com/article/10.3390/foods13101428/s1. Figure S1: The total ion chromatogams (TICs) of QC in celery seeds detected via GC-MS. Figure S2: The quantity of volatile metabolites and non-volatile metabolites in celery seeds detected via GC-MS (A), GC-IMS (B), and LC-MS (C). Figure S3: The total ion chromatogams (TICs) of QC in celery seeds detected via LC-MS. QC = quality control samples (prepared by mixing samples); N = negative ion mode; P = positive ion mode. Figure S4: The MRM metabolite detection multi peak chart of QC in celery seeds detected via LC-MS. QC = quality control samples (prepared by mixing samples); N = negative ion mode; P = positive ion mode. Table S1: Information on production region of three celery seeds. Table S2: Volatile metabolites identified in celery seeds from three production regions based on GC-MS data. Table S3: Volatile metabolites identified in celery seeds from three production regions based on GC-IMS data. Table S4: Non-volatile metabolites identified in celery seeds from three production regions based on LC-MS data. Table S5: Differential volatile metabolites identified in celery seeds from three production regions based on GC-IMS data. Table S6: Differential volatile metabolites identified in celery seeds from three production regions based on GC-MS data. Table S7: Differential non-volatile metabolites identified in celery seeds from three production regions based on LC-MS data.
