# Supplementary material for: Investigating the Impact of Origins on the Quality Characteristics of Celery Seeds Based on Metabolite Analysis through HS-GC-IMS, HS-SPME-GC-MS and UPLC-ESI-MS/MS
Source: Foods. 2024 May 7;13(10):1428. doi: 10.3390/foods13101428 (PMC11119798; doi:10.3390/foods13101428)
Supplement: Supplementary file 1 [file foods-13-01428-s001.zip › Figure S2.pdf]

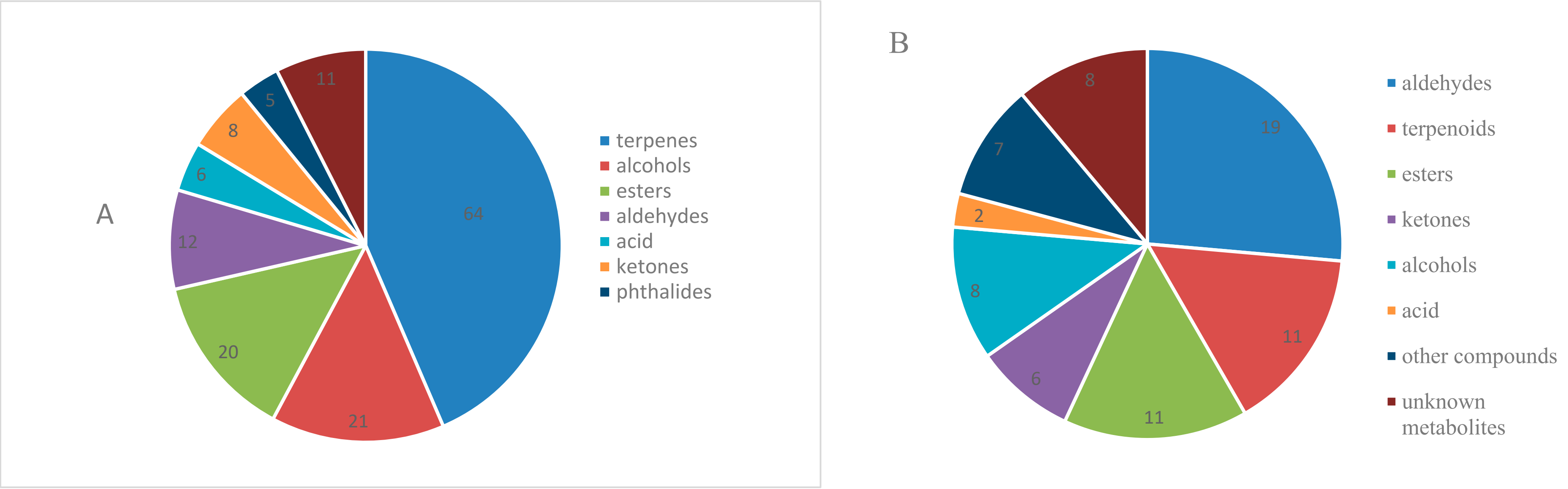
A
64
21
20
12
6
8
5
11
terpenes
alcohols
esters
aldehydes
acid
ketones
phthalides
B
19
11
11
6
8
2
7
8
aldehydes
terpenoids
esters
ketones
alcohols
acid
other compounds
unknown metabolites

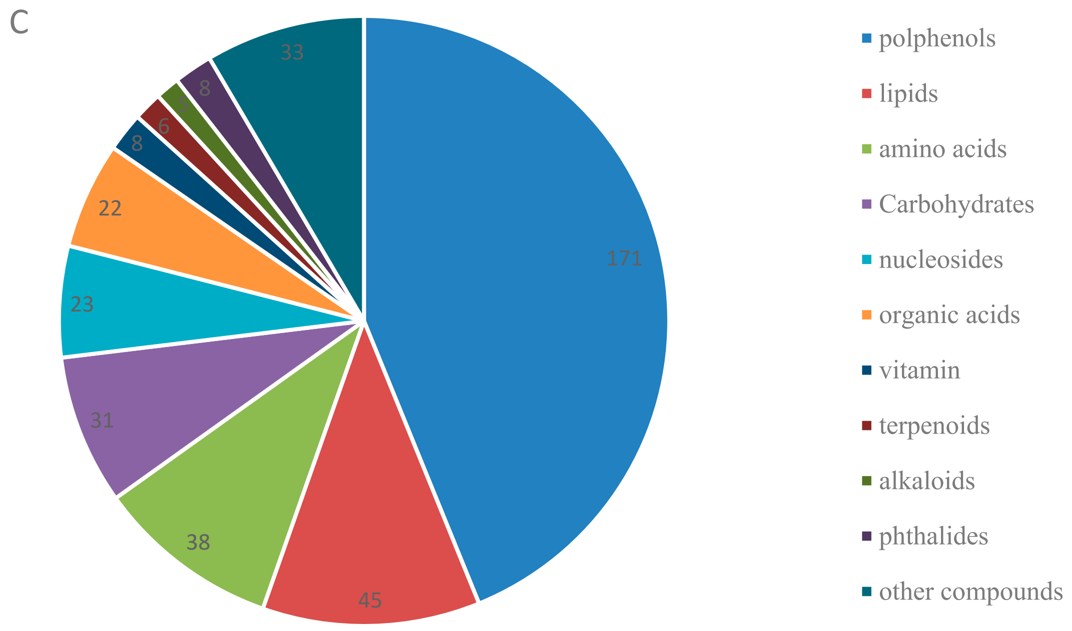

Figure. S2 The quantity of volatile metabolites and non-volatile metabolites in celery seed detected by GC-MS (A), GC-IMS (B) and LC-MS (C)
