# Supplementary material for: Investigating the Impact of Origins on the Quality Characteristics of Celery Seeds Based on Metabolite Analysis through HS-GC-IMS, HS-SPME-GC-MS and UPLC-ESI-MS/MS
Source: Foods. 2024 May 7;13(10):1428. doi: 10.3390/foods13101428 (PMC11119798; doi:10.3390/foods13101428)
Supplement: Supplementary file 1 [file foods-13-01428-s001.zip › Figure S3.pdf]

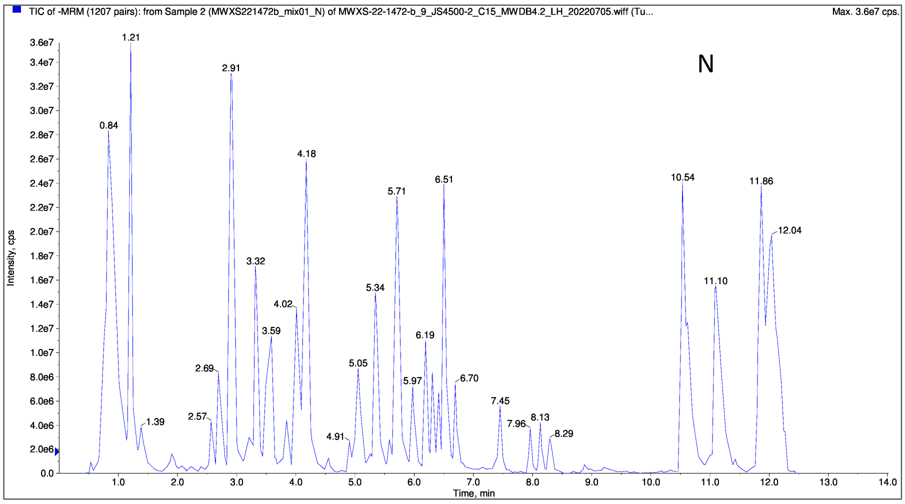

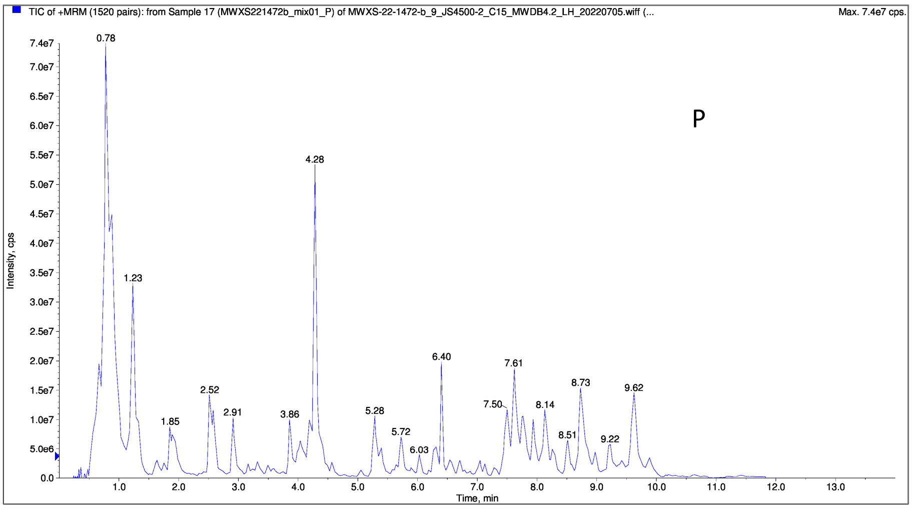

Figure S3 The total ion chromatogams (TICs) of QC in celery seed detected by LC-MS. QC Quality control samples(prepared by mixing samples), N represents negative ion mode, P represents the positive ion mode.
