# Supplementary material for: Investigating the Impact of Origins on the Quality Characteristics of Celery Seeds Based on Metabolite Analysis through HS-GC-IMS, HS-SPME-GC-MS and UPLC-ESI-MS/MS
Source: Foods. 2024 May 7;13(10):1428. doi: 10.3390/foods13101428 (PMC11119798; doi:10.3390/foods13101428)
Supplement: Supplementary file 1 [file foods-13-01428-s001.zip › Table S1.pdf]

Table S1 Information on production region of three celery seeds

NO.	Origin	Coordinate	Average annual temperature(℃)	precipitation(mm)	Climate type
HCQ	Qingxian County, Cangzhou City, Hebei Province, China	38.58N, 116.80E	12.1	618.0	Temperate semihumid continental monsoon climate
HZC	Chaling County, Zhuzhou City, Hunan Province, China	26.30N, 113.20E	17.9	1511.9	Subtropical monsoon humid climate
JJC	Chuanying District, Jilin City, Jilin Province, China	42.31N, 125.40E	4.9	700.0	Mid temperate subhumid monsoon climate
