# Supplementary material for: Investigating the Impact of Origins on the Quality Characteristics of Celery Seeds Based on Metabolite Analysis through HS-GC-IMS, HS-SPME-GC-MS and UPLC-ESI-MS/MS
Source: Foods. 2024 May 7;13(10):1428. doi: 10.3390/foods13101428 (PMC11119798; doi:10.3390/foods13101428)
Supplement: Supplementary file 1 [file foods-13-01428-s001.zip › Table S5.pdf]

Table S5 Differential volatile metabolites identified in celery seed from three production regions based on GC-IMS data

NO.	Compound	VIP	Peak Intensity [mV]		
			HCQ	HZC	JJC
IM12	Sabinene	2.570	733.04±99.34a	229.08±5.14b	37.45±5.39c
IM3	5	2.287	32.07±2.99c	458.07±6.06a	215.67±24.68b
IM25	p-Cymene	2.182	753.69±32.56a	146.05±11.39b	49.01±3.31c
IM21	β-Ocimene	2.172	46.28±2.96c	416.72±14.24a	96.46±2.36b
IM2	6	2.123	1785.37±50.67a	296.61±2.63b	123.51±2.73c
IM24	β-Phellandrene	2.064	1262.90±19.99a	807.13±11.39b	149.58±4.26c
IM36	Methyl 2-methylbutanoate-D	1.848	14.59±1.91c	118.94±1.60a	105.07±1.38b
IM4	4	1.722	106.33±5.78c	580.51±44.58b	883.32±49.47a
IM34	cis-3-Hexenyl acetate	1.684	786.88±90.89a	163.57±14.96b	96.19±5.30c
IM53	1-Octen-3-ol	1.682	79.31±5.57c	283.91±13.14b	652.21±26.87a
IM69	(E)-2-Hexenal-D	1.643	37.34±9.47c	94.52±10.17b	265.49±23.49a
IM22	Linalool	1.557	83.82±12.24b	202.75±16.87a	49.64±2.72c
IM38	Methyl butanoate	1.520	99.54±7.80b	283.06±13.00a	75.03±2.73c
IM61	1-Hexanol	1.511	342.71±5.42b	356.16±11.92a	92.98±2.78c
IM58	3-Methyl-1-butanol-D	1.481	1665.57±8.95a	336.15±3.80b	296.34±6.55c
IM35	Methyl 2-methylbutanoate-M	1.454	133.87±4.79b	671.82±3.69a	689.58±29.41a
IM13	Myrcene-T	1.433	4219.36±406.11a	869.25±44.18b	822.90±19.06b
IM71	Butanal	1.415	7106.06±81.19a	1818.13±40.39c	5058.44±34.68b
IM23	Carveol	1.354	1366.08±3.68a	428.12±39.03c	1257.05±16.35b
IM28	Dimethyl sulfide	1.335	1532.23±149.65a	388.17±17.34b	304.48±9.78c
IM72	3-Methylbutanal	1.316	4112.21±78.80a	1258.19±8.81c	3474.95±11.72b
IM39	Isoamyl acetate	1.234	79.18±11.08b	210.70±3.01a	77.45±13.47b
IM57	3-Methyl-1-butanol-M	1.222	2349.44±22.09a	575.89±38.69c	664.76±27.30b
IM67	5-Methyl-2-furfural	1.205	897.32±50.80c	3067.67±125.5a	1376.62±31.17b
IM60	1-Pentanol-D	1.203	221.64±4.69c	765.07±15.83a	348.59±14.48b
IM16	gamma-Terpinene-M	1.189	249.28±34.86a	67.98±1.82b	62.88±2.07c
IM15	Myrcene-D	1.142	1972.89±119.26a	552.02±46.06b	550.42±8.92b
IM9	beta-Pinene-T	1.060	1840.16±426.74a	768.64±6.31b	480.40±12.89c
IM75	Dodecanal	1.044	2253.36±196.51a	1785.35±54.58b	759.57±10.72c
IM6	2	1.043	939.84±6.87a	285.32±1.13c	457.83±18.35b
IM73	Octanal-M	1.043	799.82±22.16a	236.24±3.32c	323.14±20.34b
IM68	(E)-2-Hexenal-M	1.038	367.41±6.79c	1043.72±50.88b	1306.24±35.41a
IM49	1-Penten-3-one	1.026	67.88±3.38b	212.82±11.48a	213.99±7.93a
IM55	2-Propanol-M	1.002	787.50±35.12a	255.55±6.26b	260.00±17.14b
