# Supplementary material for: Investigating the Impact of Origins on the Quality Characteristics of Celery Seeds Based on Metabolite Analysis through HS-GC-IMS, HS-SPME-GC-MS and UPLC-ESI-MS/MS
Source: Foods. 2024 May 7;13(10):1428. doi: 10.3390/foods13101428 (PMC11119798; doi:10.3390/foods13101428)
Supplement: Supplementary file 1 [file foods-13-01428-s001.zip › Table S6.pdf]

Table S6 Differential volatile metabolites identified in celery seed from three production regions based on GC-MS data

NO.	Compound	VIP	Peak area		
			HCQ	HZC	JJC
XMW0709	1(7), 8-p-Menthadiene	3.004	7.47±0.31b	23.48±1.62a	1.54±0.29c
KMW0582	β-Guaiene	2.793	4847.71±196.71a	3573.87±461.87b	352.56±49.58c
KMW0210	Sabinene	2.483	34.63±0.92a	6.09±0.21b	4.30±0.03c
XMW0127	p-Cymene	2.433	923.90±32.44a	887.27±59.41a	105.48±7.77b
KMW0556	β-Selinene	2.373	422.28±21.40a	376.95±46.40a	48.58±0.83b
w38	β-Cedrene	2.244	74.63±2.89b	171.21±25.26a	22.24±3.90c
KMW0148	α-Pinene	2.228	651.91±21.85b	688.12±3.35a	94.77±6.63c
KMW0267	Benzoic acid, methyl ester	2.221	3.18±0.26c	4.16±0.11b	26.05±0.99a
WMW0050	dihydro-Actinidiolide	2.213	14.22±0.72a	15.28±2.71a	2.13±0.27b
KMW0175	(Z)-butylidene-phthalide	2.170	1589.10±18.03a	979.84±45.06b	187.19±5.05c
KMW0247	β-Phellandrene	2.081	18994.39±892.85a	5777.22±216.73b	2562.34±144.44c
KMW0193	β-Pinene	2.041	3235.60±380.78b	4996.25±180.20a	765.57±168.84c
XMW1401	Decanoic acid, ethyl ester	1.948	20.58±5.08a	4.45±0.70c	7.41±0.82b
KMW0329	Camphor	1.946	318.43±24.77a	69.46±2.63c	102.32±5.70b
XMW0780	Cosmene	1.889	41.62±3.40a	18.12±1.45b	6.25±0.66c
KMW0531	(E,Z)-3,6-Nonadien-1-ol	1.717	38.07±2.0c	130.36±1.53b	154.49±11.98a
NMW0075	γ-Terpineol	1.700	16.82±2.33b	6.27±0.55c	25.18±1.66a
KMW0547	Carveol acetate	1.663	12.88±1.49b	8.32±0.06c	39.07±1.25a
XMW0228	1-ethenyl-Cyclohexanol	1.636	54.78±1.29a	16.54±1.15c	43.09±2.61b
D186	trans-Carveol	1.552	47.96±3.38a	14.27±1.51c	19.26±2.29b
KMW0545	Dodecanal	1.501	9.59±0.83b	28.89±0.28a	30.10±0.92a
XMW0410	1-Nonen-4-ol	1.494	2.97±0.46a	1.16±0.13b	3.61±0.25a
KMW0159	1-Heptanol	1.487	8.44±1.47c	20.02±0.02b	34.36±1.42a
NMW0052	9-Hexadecenoic acid	1.447	113.84±1.44c	303.28±3.24b	401.66±4.45a
WMW0040	trans-β-Ocimene	1.417	14.38±0.61b	32.50±2.01a	10.23±0.57c
XMW0146	trans-Sedanolide	1.414	4525.21±272.17a	2435.43±469.26b	1095.74±28.91c
NMW0093	Vanillin	1.399	8.46±0.38c	12.71±0.43b	34.43±0.06a
XMW0006	Globulol	1.392	31.73±0.08b	15.32±0.97c	50.44±1.04a
XMW0574	Hexanoic acid, ethyl ester	1.358	3.05±0.02b	8.73±0.56a	7.33±0.82a
KMW0304	1,3,8-p-Menthatriene	1.338	5.13±0.73c	8.21±0.51b	19.73±1.62a
XMW0306	Heptanoic acid, ethyl ester	1.321	36.13±3.76b	20.22±0.02c	66.12±2.31a
KMW0173	1-Octen-3-ol	1.202	1.49±0.13c	2.58±0.11b	4.97±0.33a
KMW0558	Linalyl butyrate	1.176	117.21±4.89b	88.69±0.52c	265.05±39.05a
NMW0123	cis-1,3,trans-1,4-p-Menthane-3,8-diol	1.131	8.56±0.82a	3.53±0.28c	4.82±0.87b
NMW0084	Cyclopentacycloheptene	1.127	16.12±0.20a	6.80±0.47b	7.49±0.85b
KMW0386	cis-Dihydrocarvone	1.082	45.61±3.43a	23.99±0.30b	16.33±0.85c
KMW0199	β-Myrcene	1.074	2475.36±48.63a	1582.63±102.14b	839.48±37.54c
XMW0659	3-n-Butylphthalide	1.040	8.74±0.76a	5.17±0.04b	3.12±0.32c
NMW0773	Cyclohexanone	1.036	1.69±0.16c	3.63±0.04b	3.73±0.17a
KMW0589	Humulene	1.001	4.17±0.02c	8.20±1.30b	10.11±0.66a
