# Supplementary material for: Investigating the Impact of Origins on the Quality Characteristics of Celery Seeds Based on Metabolite Analysis through HS-GC-IMS, HS-SPME-GC-MS and UPLC-ESI-MS/MS
Source: Foods. 2024 May 7;13(10):1428. doi: 10.3390/foods13101428 (PMC11119798; doi:10.3390/foods13101428)
Supplement: Supplementary file 1 [file foods-13-01428-s001.zip › Table S7.pdf]

Table S7 Differential non-volatile metabolites identified in celery seed from three production regions based on LC-MS data

NO.	Compound	VIP	Peak area		
			HCQ	HZC	JJC
pmp000344	3',4',7-Trihydroxyflavone	5.106	37510.8±5773.7a	1877.6±70.66b	84.24±3.42c
MWSHY0189	Apigenin-7-O-glucoside	3.834	63896.8±5909.7a	2588.6±415.1b	789.2±24.0c
Hmqp003435	Apigenin	3.783	96754.9±8765.8a	85963.5±5694.0a	2747.9±211.4b
mws0063	Genistein	3.426	247.5±5.4b	649.05±49.1a	25.4±3.9c
mws0183	Protocatechuic acid	3.175	705.6±32.8a	187.2±6.53b	18.2±3.1c
pmb3012	Chrysoeriol-7-O-glucoside	3.101	1557.1±129.24b	262.5±23.8c	4157.9±2.84a
pmb0608	Cinnamic acid	3.058	97.28±13.53c	1940.9±119.6a	663.4±34.8b
pme1439	p-Coumaric acid	3.039	277.9±31.3b	2471.4±137.7a	203.2±12.6c
Xmyp005654	Kaempferol-4'-O-glucoside	2.904	7962.4±661.4b	71024.7±7314.5a	7130.0±135.9c
Lmjp004941	3,5,4'-Trihydroxy-7-methoxyflavone	2.853	59205.9±4064.7a	2503.6±149.9c	8732.8±15.1b
MWS20194	Chrysoeriol-7-O-(6''-malonyl)glucoside	2.697	2638.9±326.5a	423.3±22.9b	105.4±15.2c
Zmyn004548	12-Oxo-phytodienoic acid	2.654	36.0±2.59a	2.78±0.1b	2.62±0.66b
MWS20143	Luteolin	2.634	2304.1±198.43a	239.4±26.5b	119.6±6.66b
MWS20151	Apigenin-7-O-(2''-O-apiosyl)(6''-Malonyl)glucoside	2.616	6887.8±278.6a	782.7±66.11b	343.3±5.8c
Zmxp002867	Chrysoeriol-5,7-di-O-glucoside	2.615	281.6±31.4a	31.2±5.6b	14.2±1.7c
pmn001367	Protocatechuic acid-4-O-glucoside	2.592	915.2±25.7a	974.7±67.1a	88.8±2.8b
MWSHY0104	Luteolin-7-O-glucoside	2.556	28900.8±5331.6a	2463.5±156.5b	2212.2±74.2c
Lmmp003817	Kaempferol-3-O-(6''-malonyl)glucoside	2.524	1935.8±61.8a	1884.3±125.3a	186.9±9.3b
mws2212	Caffeic acid	2.451	3258.6±118.0a	924.8±44.3b	180.9±26.3c
Lmlp012720	Dibutyl phthalate	2.433	9858.9±452.8a	10438.1±434.5a	1100.7±31.8b
Hmap010205	umbelliprenin	2.345	280.3±23.1a	225.2±16.3b	27.9±3.0c
pmp000585	Apigenin-7-O-(6''-malonyl)glucoside	2.339	158.9±7.2a	93.2±3.6b	13.1±1.5c
Lmjp003655	6-C-MethylKaempferol-3-glucoside	2.276	60950.2±3493.7a	7114.2±157.8b	5751.1±142.8c
Hmmp004965	Diosmetin-7-O-glucoside	2.236	46315.6±4440.7a	6788.3±499.3b	3680.1±151.5c
mws1155	D-Mannitol	2.194	137.0±11.1b	143.7±11.5b	1486.0±77.6a
Lmyn007883	9,16-Dihydroxypalmitic acid	2.156	515.9±14.7a	67.5±11.3b	56.3±2.7b
Zmbp004146	Praeroside IV	2.132	103.0±12.9b	197.2±20.5a	26.3±1.2c
Hmqp002870	Luteolin-7-O-Sophoroside-5-O-arabinoside	2.128	23.6±4.1b	124.0±8.6a	24.4±2.7b
MWS20148	Apigenin-4'-O-glucoside*	2.121	9329.8±147.9a	2208.1±112.0b	741.0±60.4c
Zmyn004676	17-Hydroxylinolenic acid	2.052	1121.3±79.1a	149.3±4.4b	163.8±20.3b
HJAP148	Kaempferol-3-O-sambubioside	2.031	1296.4±137.6a	208.5±27.1b	140.5±7.3c
mws0014	Ferulic acid	1.924	954.5±32.9a	156.8±39.8b	132.7±10.1b
MWSHY0046	Quercetin-3-O-glucoside	1.883	31.6±4.9c	203.6±39.1a	90.5±2.2b
pmf0472	Apiin	1.862	1032.0±48.2a	193.9±14.9b	133.9±11.5c
pmp001235	Cnidilide	1.724	2080.2±99.9a	943.5±166.9b	278.1±23.7c
MWSmce692	γ-Linolenic Acid	1.719	314.5±45.2a	80.4±7.6b	42.1±5.5c
pmb0571	Apigenin-7-O-(2''-glucosyl)arabinoside	1.688	2541.3±135.0a	531.5±99.7c	1367.5±18.9b
Hmcn002875	6-Hydroxyluteolin	1.645	159.3±16.9a	45.1±4.3b	161.5±6.2a

pme0519	D-Sucrose	1.629	3178.3±265.6b	3104.1±424.9b	13845.9±557.2a
Lmbp002962	Isofraxetin	1.578	79.0±9.8a	82.8±9.7a	19.1±0.3b
MWSCX014	Scopoletin	1.529	778.3±57.2a	189.0±28.7b	152.0±21.5b
pmb0464	L-Aspartic acid-O-diglucoside	1.502	7446.6±123.6b	6414.9±215.2c	26323.7±581.5a
pme3011	γ-Aminobutyric acid	1.479	12314.2±641.3a	4737.2±117.5b	2106.5±66.2c
mws1164	D-Fructose	1.467	786.8±20.9b	688.6±18.5c	2722.8±55.0a
Hmcp002123	Isoscopoletin	1.465	606.5±26.4a	176.3±27.3b	113.1±17.9c
mws0281	Citric Acid	1.463	1430.6±49.2c	5177.0±179.3b	7104.4±255.1a
Lmzp002365	Hesperetin-7-O-glucoside	1.462	1719.7±233.7a	481.2±12.0b	330.7±15.8c
mws0367	α-Linolenic Acid	1.456	69645.6±2378.4a	21557.9±1198.1b	12773.9±75.6c
mws0178	Chlorogenic acid	1.450	91065.9±399.1a	21374.1±2330.3c	34225.0±1288.2b
Lmhp001461	L-Prolyl-L-Leucine	1.434	771.8±69.5a	212.4±5.2c	522.7±13.4b
Zmpn000199	D-Galactaric acid	1.405	2110.6±93.8c	2303.6±50.1b	8170.5±120.8a
mws2125	Phosphoenolpyruvate	1.400	51.9±3.5c	69.2±7.9b	225.7±20.4a
HX1363	isoscopoletin	1.395	669.6±74.3a	190.3±10.4b	145.1±5.8c
Lmbn001981	2,5-Dihydroxybenzaldehyde	1.381	470.3±58.2a	123.7±20.0b	120.7±12.4b
mws1179	Naringenin-7-O-glucoside	1.365	246.7±8.9a	64.3±3.7b	66.5±3.2b
Zmhp003514	6,7,8-Tetrahydroxy-5-methoxyflavone	1.348	1266.7±84.9a	704.2±41.2b	264.7±3.2c
Lmbp102601	7-Hydroxycoumarin	1.340	563.3±67.1b	796.3±42.2a	223.9±27.1c
MWSHY0009	Diosmetin	1.318	9239.9±546.8a	2516.2±174.5b	2730.5±48.9b
mws0376	Fumaric acid	1.310	189.4±5.1a	195.0±21.1a	58.3±3.1b
MWSmce324	Psoralen	1.308	232.6±17.9a	66.2±5.6c	93.5±4.8b
pme0253	N-Acetyl-L-leucine	1.302	152.5±16.7a	47.9±8.8b	35.9±6.5b
mws0369	Arachidonic Acid	1.281	10.2±1.5c	28.7±6.4b	45.9±1.2a
Hmhp001812	2'-O-Methyladenosine	1.279	1013.7±90.9a	377.4±52.5b	229.6±31.4c
MWS1926	DL-Leucine	1.267	8565.7±418.7b	6301.1±192.8c	20817.4±376.6a
Lmmn001643	2-Hydroxycinnamic acid	1.265	3812.2±137.8a	1174.3±95.5b	1016.9±38.7c
Hmqp005455	15(R)-Hydroxylinoleic Acid	1.248	263.1±26.3a	86.0±7.7c	188.6±30.8b
pme3033	N,N-Dimethylglycine	1.216	8585.4±569.4a	3880.1±146.5b	2009.4±32.9c
Smhn002274	Blumenol C glucoside	1.215	93.1±4.6b	103.5±5.3b	306.8±8.3a
MWSHY0145	Eriodictyol	1.213	352.5±12.1b	891.2±22.2a	347.6±10.8b
pme0490	Vitamin B3	1.188	787.9±119.6a	276.3±54.6b	208.3±8.5b
MWSmce083	Ferulic acid methyl ester	1.167	12.1±0.7c	36.3±1.5a	19.1±0.9b
Lmhp112042	1-Linoleoylglycerol	1.143	204.0±15.6c	628.2±93.1a	369.2±25.0b
mws0639	2,3-Dihydroxybenzoic Acid	1.132	499.7±16.4a	168.9±15.7c	306.7±86.3b
Lbhp011920	Scopoletin 7-O-β-D-Sophoroside	1.128	390.0±1.8a	214.3±8.6b	103.8±18.0c
pmb3074	5-O-p-Coumaroylquinic acid	1.117	33714.6±913.2a	11059.3±342.0b	12795.6±1439.5b
Zmhn002334	6-O-Feruloyl-β-D-glucose	1.100	21.5±1.1b	64.6±7.5a	58.6±9.8a
pme0271	Maleic acid	1.099	169.4±15.3b	225.0±21.3a	69.4±5.6c
mws1611	Z-Ligustilide	1.096	551.7±22.2a	231.8±27.9b	526.9±27.6a
mws0866	D-Glucose 6-phosphate	1.085	1938.1±231.3b	1149.0±12.4c	3133.4±83.9a
mws0214	D-Sorbitol	1.083	348.7±35.3c	618.9±30.7b	1253.6±7.0a
pme1474	5'-Deoxy-5'-(methylthio)adenosine	1.078	1606.4±144.5c	3956.8±80.8b	5527.4±124.9a
mws0668	Xanthosine	1.070	1393.3±71.5a	592.9±51.0b	403.2±26.9c

pmf0397	Arachidic acid	1.065	4424.7±132.2a	1740.4±121.9b	1353.6±204.4c
pme1178	Guanosine	1.038	8142.3±746.9a	2995.9±139.6b	2885.8±170.1b
MWS2442	D-Fructose 6-Phosphate	1.035	1682.6±125.4b	1285.2±120.7c	3417.8±105.1a
MWSHY0017	Naringenin	1.028	159.6±17.0a	64.8±7.3b	54.2±1.1c
pmp000256	Angelicide	1.014	165.2±8.5a	63.0±2.2b	57.8±5.6b
