# Supplementary figures and images for: Investigating the Impact of Origins on the Quality Characteristics of Celery Seeds Based on Metabolite Analysis through HS-GC-IMS, HS-SPME-GC-MS and UPLC-ESI-MS/MS
Source: Foods. 2024 May 7;13(10):1428. doi: 10.3390/foods13101428 (PMC11119798; doi:10.3390/foods13101428)

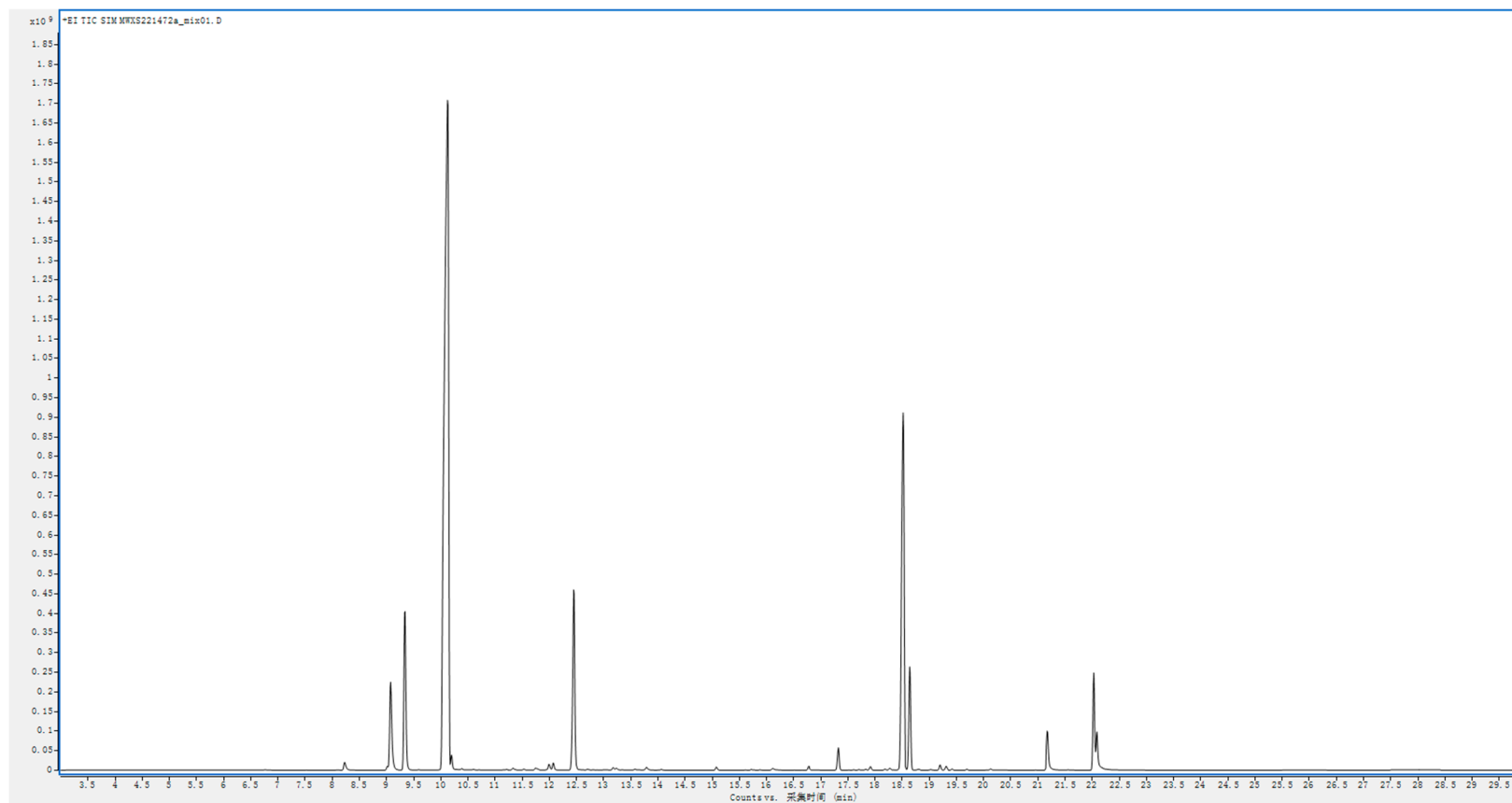

Figure S1 The total ion chromatograms (TICs) of QC in celery seed detected by GC-MS

Supplement: Supplementary file 1 [file foods-13-01428-s001.zip › Figure S1.pdf]
